# Agreement and repeatability of vascular reactivity estimates based on a breath-hold task and a resting state scan

**DOI:** 10.1016/j.neuroimage.2015.03.004

**Published:** 2015-06

**Authors:** Ilona Lipp, Kevin Murphy, Xavier Caseras, Richard G. Wise

**Affiliations:** a Cardiff University Brain Research Imaging Centre (CUBRIC), School of Psychology, Cardiff University, UK; b MRC Centre for Neuropsychiatric Genetics and Genomics, Institute of Psychological Medicine and Clinical Neurosciences, Cardiff University, UK

**Keywords:** Vascular reactivity, Breath-hold, Resting state, RSFA, Repeatability

## Abstract

FMRI BOLD responses to changes in neural activity are influenced by the reactivity of the vasculature. By complementing a task-related BOLD acquisition with a vascular reactivity measure obtained through breath-holding or hypercapnia, this unwanted variance can be statistically reduced in the BOLD responses of interest. Recently, it has been suggested that vascular reactivity can also be estimated using a resting state scan.

This study aimed to compare three breath-hold based analysis approaches (block design, sine–cosine regressor and CO_2_ regressor) and a resting state approach (CO_2_ regressor) to measure vascular reactivity. We tested BOLD variance explained by the model and repeatability of the measures. Fifteen healthy participants underwent a breath-hold task and a resting state scan with end-tidal CO_2_ being recorded during both. Vascular reactivity was defined as CO_2_-related BOLD percent signal change/mm Hg change in CO_2_.

Maps and regional vascular reactivity estimates showed high repeatability when the breath-hold task was used. Repeatability and variance explained by the CO_2_ trace regressor were lower for the resting state data based approach, which resulted in highly variable measures of vascular reactivity.

We conclude that breath-hold based vascular reactivity estimations are more repeatable than resting-based estimates, and that there are limitations with replacing breath-hold scans by resting state scans for vascular reactivity assessment.

## Introduction

The blood-oxygenation-level-dependent (BOLD) signal that is acquired during functional magnetic resonance imaging (fMRI) is commonly used as a measure of neural activity in the brain. The nature of the BOLD signal makes it susceptible not only to changes in neural activity but also depends in part on the reactivity of the cerebro-vascular system (for a review see Logothetis and Wandell, 2004). One aspect of this vascular reactivity can be measured by changing the CO_2_ content of the blood; CO_2_ being a vasodilator. It has been shown that natural fluctuations in CO_2_ during a resting state scan coincide with low frequency fluctuations in the BOLD signal (Wise et al., 2004). Furthermore, the BOLD signal following an increase in CO_2_ in a region can explain up to 50% of variance between participants in task-related BOLD responses in that region (Kannurpatti et al., 2012; Liu et al., 2013). This confound can cause problems for studies investigating task-dependent BOLD signals, in particular if group differences in vascular reactivity exist, for example, in the case when comparing older and younger participants (Handwerker et al., 2007; Riecker et al., 2003; Thomason et al., 2005).

One approach to this problem is to estimate vascular reactivity in a separate scan and to set up a statistical design that controls for regional or inter-individual differences in vascular reactivity (Murphy et al., 2011). This can reduce unwanted variability between participants, leaving variability that better reflects differences in the neural responses (Liu et al., 2013; Murphy et al., 2011; Thomason et al., 2007). A common way to estimate vascular reactivity is to increase CO_2_ by inducing hypercapnia through respiratory challenges (artificially regulating the CO_2_ level of the environment) or through breath-hold tasks. With both of these methods, the CO_2_ level and as a result the cerebral blood flow (CBF) can be modulated, under the assumption of an unchanged rate of oxygen consumption (Poulin et al., 1996). When breath-hold or respiratory challenges are used to estimate vascular reactivity, the increase in CO_2_ leads to wide-spread BOLD responses throughout all of grey matter (e.g. Bandettini and Wong, 1997).

Increasing inspired CO_2_ and breath-holding are the most commonly used approaches to measure vascular reactivity, and Kastrup et al. (2001) showed that both yield comparable results. However, there are some problems related to the two methods. Using the respiratory challenge approach is logistically challenging, and not all participants are compliant with the breathing apparatus. Breath-holds are more easily implemented, however, participants may vary in their ability to hold their breath. One way to compensate for performance differences is to record the end-tidal CO_2_ and use the traces for modelling the BOLD response (Bright and Murphy, 2013). Another way of assessing vascular reactivity avoiding this problem has been proposed by Kannurpatti and Biswal (2008). They define a resting state physiological fluctuation amplitude (RSFA) which is calculated as the temporal standard deviation of the BOLD time series obtained during a resting state scan. This method has been used to scale task-related BOLD responses (e.g. Kannurpatti et al., 2011), and Kannurpatti and Biswal (2008) found high similarity between RSFA maps and maps showing the temporal standard deviation of the BOLD time series obtained during breath-hold or a 5% CO_2_ challenge. However, it is not clear whether RSFA is a specific measure of vascular reactivity, since it is ignorant of underlying changes in CO_2_ and undoubtedly incorporates BOLD signal changes originating from fluctuations in neural activity rather than purely vasoactive stimulation. During a breath-hold scan the changes in CO_2_ are induced and happen during specified periods of time. This gives the researcher several options for modelling the BOLD data. Murphy et al. (2011) compared a number of models with regard to how well they fit a BOLD-time course obtained during 6 cycles of 20 second breath-holds. The simplest model is a block model convolved with the hemodynamic response function (HRF). Another option is a sine–cosine waveform regressor at the task frequency. Using these two models, one assumes that participants follow task instructions and can manage to hold their breath for the specified amount of time. A way to avoid this assumption is to model the end-tidal CO_2_ measured during the scan convolved with the HRF. Murphy et al. (2011) found that the sine–cosine model outperforms the block design and that using the recorded CO_2_ trace regressor leads to a better model fit than using the sine–cosine regressors, but not in all brain regions. However, when participants vary in their breath-hold performance, the CO_2_ regressor can substantially improve the quality of vascular reactivity estimations (Bright and Murphy, 2013).

If end-tidal CO_2_ is acquired during a resting state scan, the CO_2_ regressor approach could in principle also be used for resting scan data to evaluate vascular reactivity. This permits the examination of CO_2_ related BOLD fluctuations and could lead to a more specific measure of vascular reactivity during rest than the temporal standard deviation of the BOLD signal, potentially reducing the confounding effect of fluctuations in neural activity contributing to a vascular reactivity estimate. In this study, we measured end-tidal CO_2_ during a breath-hold task and a resting state scan to obtain scaled BOLD signal changes as vascular reactivity measures (CO_2_-related percent change in BOLD/mm Hg CO_2_; also see Wise et al., 2004). This enabled us to investigate whether the breath-hold approach and the resting state approach result in measures of vascular reactivity, and how well the model established using the CO_2_ traces fits the BOLD data.

The model fit gives an indication of the quality of a method used. Another quality index is the repeatability of the obtained results. In a number of studies different ways of breath-hold acquisition are compared with regard to repeatability as measured by inter-trial variability (Magon et al., 2009; Scouten and Schwarzbauer, 2008; Thomason and Glover, 2008). Comparing inter-trial and inter-individual variance over different conditions makes it possible to define the most repeatable condition. The intraclass correlation coefficient (ICC) reflects the ratio between the data variance of interest (between-participant differences) and the total data variance (Shrout and Fleiss, 1979), and has frequently been applied to fMRI data (Bright and Murphy, 2013; Caceres et al., 2009). The ICC can be applied to extracted percent signal change from an area of interest, as well as to voxels in order to obtain an estimate of spatial repeatability (repeatability of the signal spatial distribution; e.g. Lipp et al., 2014). Magon et al. (2009) applied a similar approach to breath-hold data, calculating Pearson's correlation coefficient over the *t* values of all voxels in the grey matter. Mean correlation in the 11 participants for a 21 second long breath-hold was .70, which indicates good repeatability (Cicchetti, 2001). Recently, Bright and Murphy (2013) reported high repeatability of spatial maps as well as locally extracted averages of vascular reactivity when end-tidal CO_2_ regressors were used to analyse breath-hold data. In addition to the ICC, the coefficient of variation (CV) is an often used measure of repeatability, distinguishing variance between participants (CV_between_), and variance within participants (CV_within_), while the ICC depends on both these components and is therefore susceptible to the variability in the investigated sample.

The aim of this study was to compare the vascular reactivity estimates obtained during breath-holding and during rest on three levels: the estimates in %SC/mm Hg CO_2_, the model fit of the CO_2_ regressor and between-session repeatability of the vascular reactivity maps and regionally averaged vascular reactivity. To allow direct comparison to previous studies, we also included measures of temporal standard deviation (RSFA and temporal standard deviation of the BOLD time series during breath-holding) in the analysis. Furthermore, we aimed to investigate whether between-session repeatability is influenced by the model used to analyse the breath-hold data (block design vs. sine–cosine regressor vs. CO_2_ regressor). An additional aim of this study was to establish the minimum number of breath-hold cycles needed to obtain repeatable vascular reactivity maps.

## Methods

### Sample

Fifteen (8 male) participants with a mean age of 24 (range: 21–28) voluntarily took part in the study. They underwent the same scanning protocol twice with a mean interval of 23 days (range: 15–34). One participant was excluded from analysis due to problems with the acquired CO_2_ trace. All participants gave written consent. The study was approved by the Cardiff University School of Medicine Research Ethics Committee.

### Breath-hold task

The breath-hold task was adapted from Murphy et al. (2011). During the task, breathing instructions were presented on the screen, guiding the participant through 6 cycles of breath-holding and recovery, each with four different phases: paced breathing (alternating breathing in and breathing out for 3 s each) for 18 s, end-expiration breath-holding for 15 s, exhalation, and final recovery (spontaneous breathing with no breathing instructions) for 15 s. The task took 5 min to complete. End-expirational breath-hold was chosen because it has been shown that a shorter breath-hold duration is needed to obtain the same signal changes, and because the inspiration before a breath-hold varies between participant with regard to depth and intrathoracic pressure which introduces additional variability (Kastrup et al., 1998; Thomason and Glover, 2008).

### Image acquisition

The participants underwent gradient-echo echo-planar imaging at 3 T (GE HDx MRI System) with a T2* weighted imaging sequence (TR = 3 s, TE = 35 ms, receive-only head coil). 140 volumes were acquired during the resting state period, 108 during the breath-hold task. The breath-hold task was presented using Presentation (Neurobehavioral Systems, Albany, CA) and rear-projected onto a screen behind the participant's head that was visible through a mirror mounted on the head RF coil. The orientation of the axial slices was parallel to the AC–PC line. During the resting state period, participants were presented with a black screen and instructed to keep their eyes open and to relax.

A T1 weighted whole-brain structural scan was also acquired for purposes of image registration (1 × 1 × 1 mm resolution, 256 × 256 × 176 matrix size). The structural image was only acquired during session 1, and this image was used for registration for the functional images of session 1 and session 2.

### CO_2_ recordings

During both scanning sessions end-tidal carbon dioxide (PetCO_2_) and end-tidal oxygen (PetO_2_) were recorded using a nasal cannula attached to rapidly responding gas analysers (AEI Technologies, PA) to provide representative measures of changes in arterial partial pressures of both gases.

### Image data analysis

The acquired data were preprocessed using FEAT (FMRIB Expert Analysis Tool, v5.98, www.fmrib.ox.ac.uk/fsl, Oxford University, UK). Preprocessing steps before model fitting were applied to each participant's time series, and included: highpass filtering of the data (100 s temporal cutoff), non-brain removal using BET (Smith, 2002), MCFLIRT motion correction (Jenkinson et al., 2002), spatial smoothing with a Gaussian kernel of full-width-half-maximum 5 mm and fieldmap-based EPI unwarping using PRELUDE + FUGUE (Jenkinson, 2003, 2004); for one person this was not performed due to problems during the acquisition of the fieldmaps. Functional images were registered using FLIRT (Jenkinson and Smith, 2001) in a first step to the structural image with 6 degrees of freedom, and in the second step to the Montreal Neurological Institute (MNI) space with 12 degrees of freedom. First-level analysis of the breath-hold task was performed in three different ways using different sets of regressors: 1) a boxcar regressor with a lag of 9 s (Murphy et al., 2011), 2) a sine and cosine wave at the task frequency (0.02 Hz) and 3) the recorded CO_2_ trace (HRF convolved) including a temporal derivative (Murphy et al., 2011). The resting state data were analysed with the recorded CO_2_ (convolved with a HRF) trace as a regressor, also including a temporal derivative. The CO_2_ traces were temporally filtered by FEAT for both, the breath-hold and the resting state analysis. We will refer to these approaches as *BH*_Block_, *BH*_Sine−cosine_, *BH*_CO2_ and *Resting*_CO2_.

### RSFA calculation

To create RSFA maps, the temporal standard deviation of the BOLD time series was calculated for each voxel (Kannurpatti and Biswal, 2008) for the resting state data. This was performed after all the preprocessing steps described above. To be able to compare results to previous studies, the temporal standard deviation of the BOLD time series was also calculated for the breath-hold scan. We will refer to these approaches as *Resting*_RSFA_ and *BH*_Tsd_.

### Estimation of the model fit

The coefficient of determination (R^2^) was used as an estimation of how well the applied model fits the data. This measure gives an indication of the amount of variance in the time series explained by the applied model. For each of the analysis methods and each participant, an R^2^ map was calculated by applying the following equation to each voxel:

$$\frac{MSS_{\mathrm{time}‐\mathrm{series}}-MSS_{\mathrm{residuals}}}{MSS_{\mathrm{time}‐\mathrm{series}}}\text{,}$$

whereby the mean sum of squares (MSS) was calculated by squaring and temporally summing over the demeaned time series and residual time series, respectively. For each participant, the median R^2^ was extracted from grey matter over the whole brain as well as for grey matter within a number of anatomical masks selected from the WFU-PickAtlas (Version 3.0.4, Wake Forest University, School of Medicine, Winston-Salem, North Carolina, www.ansir.wfubmc.edu).

### Calculation of the BOLD percent signal change/mm Hg CO_2_

For each analysis method, the residuals after model fitting were subtracted from the preprocessed time series in order to obtain a model-fitted time series. To estimate the BOLD signal change per unit change of CO_2_, the robust range of this fitted BOLD time series was divided by the temporal mean and multiplied by 100 (to get % signal change), and then divided by the robust range of the HRF convolved CO_2_ trace (to get % signal change/mm Hg). Note that this CO_2_ range was the same for all three breath-hold based methods. In order to minimise the risk of outliers, a robust range was defined as the absolute difference between the 10th percentile and the 90th percentile. The median signal change was then extracted for the grey matter mask and for the additional anatomically defined regions.

For all vascular reactivity maps (*BH*_Block_, *BH*_Sine−cosine_, *BH*_CO2_, *Resting*_CO2_, *Resting*_*RSFA*_ and *BH*_Tsd_), voxels were excluded if they were outside the brain (using AFNI function 3dSkullStrip) and if they had a negative *z* value in the original Feat analysis (indicating “negative vascular reactivity”; this step could not be done for *Resting*_*RSFA*_ and *BH*_Tsd_).

### Statistical analysis

#### Repeatability analysis

The intraclass correlation coefficient (ICC([3,1]); Shrout and Fleiss, 1979) was used as a measure of repeatability. ICCs were interpreted according to commonly used guidelines (Cicchetti, 2001; see also e.g. Van den Bulk et al., 2013) that classify values of < .41 as “poor”, values between .41 and .59 as “fair”, values between .60–.74 as “good” and values > .74 as “excellent”.

In order to determine the repeatability of vascular-reactivity maps, voxel-wise ICCs_spatial_ were calculated for each participant separately. This was performed using the percent signal change/mm Hg maps and all voxels in grey matter. The same approach was used to estimate the agreement between vascular-reactivity maps obtained using different analysis approaches, but for this analysis Pearson correlation coefficients (*r* spatial) were used. All repeatability analyses were performed using in-house MATLAB (MathWorks, Natick, MA) scripts.

A CV_within_ was calculated for each person by dividing the standard deviation of the two vascular reactivity measures by their mean. CV_between_ was calculated for both sessions (by dividing the standard deviation by the mean of all participants' vascular reactivity estimates) and averaged over the two sessions.

#### Statistical comparisons between analysis methods

We compared the extracted median values for R^2^, BOLD signal changes, spatial repeatability ICCs and CV_within_ for the three breath-hold based methods and the resting approach by calculating Friedman tests, using the function implemented in MATLAB (MathWorks, Natick, MA). This nonparametric test was chosen due to the nature of the values used and due to problems with the assumption for sphericity. Friedman tests were performed for comparing the three breath-hold based methods (*BH*_Block_, *BH*_Sine−cosine_, *BH*_CO2_) and for comparing the two approaches using the CO_2_ regressor (*BH*_CO2_ and *Resting*_CO2_).

## Results

**Fig. 1:**
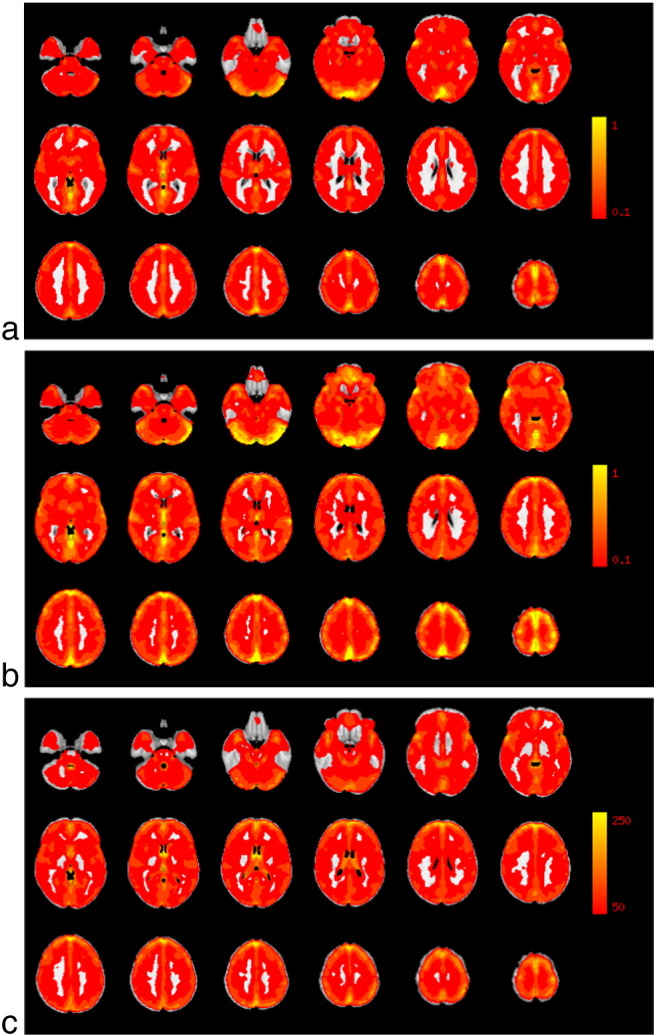
Average vascular reactivity maps, calculated with a) the *BH*_CO2_ method (in % SC/mm Hg CO_2_), b) the *Resting*_CO2_ method (in % SC/mm Hg CO_CO2_), and c) the RSFA approach (in arbitrary units). The average was calculated over 14 participants.

Average vascular reactivity maps and RSFA maps are shown in Fig. 1.

### Model fit

**Fig. 2:**
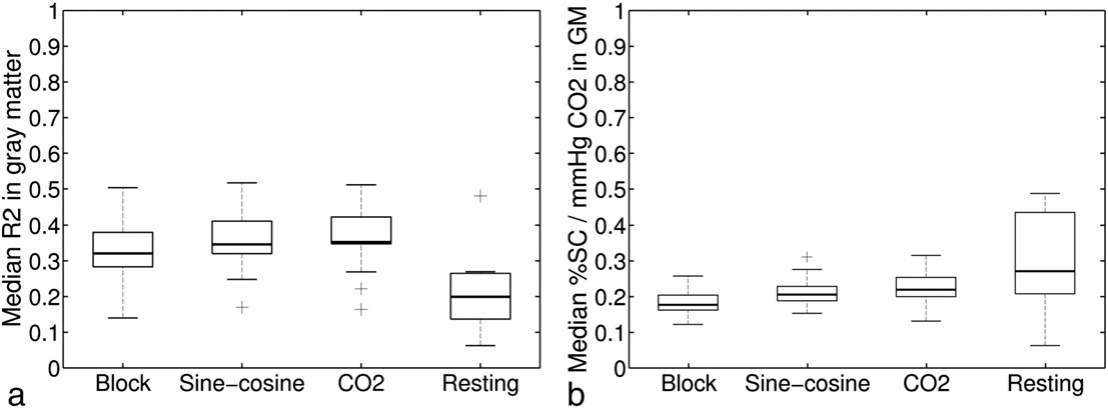
a) Comparison of the analysis methods with regard to the model fit as estimated by the median R^2^ in grey matter. b) Comparison of the analysis methods with regard to median BOLD % signal change/mm Hg CO_2_ estimations in grey matter.

In order to examine the model fit, data from session 1 was used. The amount of variance explained by the four methods was calculated for each participant. There was a significant difference in the variance explained between the breath-hold based methods (*χ*[2] = 18.1, *p* < .0001, see Fig. 2a). Pairwise comparisons revealed that the *BH*_Block_ design explained the BOLD time course significantly worse than the *BH*_Sine−cosine_ (*p* = .0006) and the *BH*_CO2_ design (*p* = .004). No significant difference was found between the *BH*_Sine−cosine_ and *BH*_CO2_ regressors (*p* > .99; all *p* values corrected for number of pairwise comparisons (3)). The model fit in the *Resting*_CO2_ set was significantly lower than in the *BH*_CO2_ approach (*χ*[1] = 7.14, *p*= .0075).

**Fig. 3:**
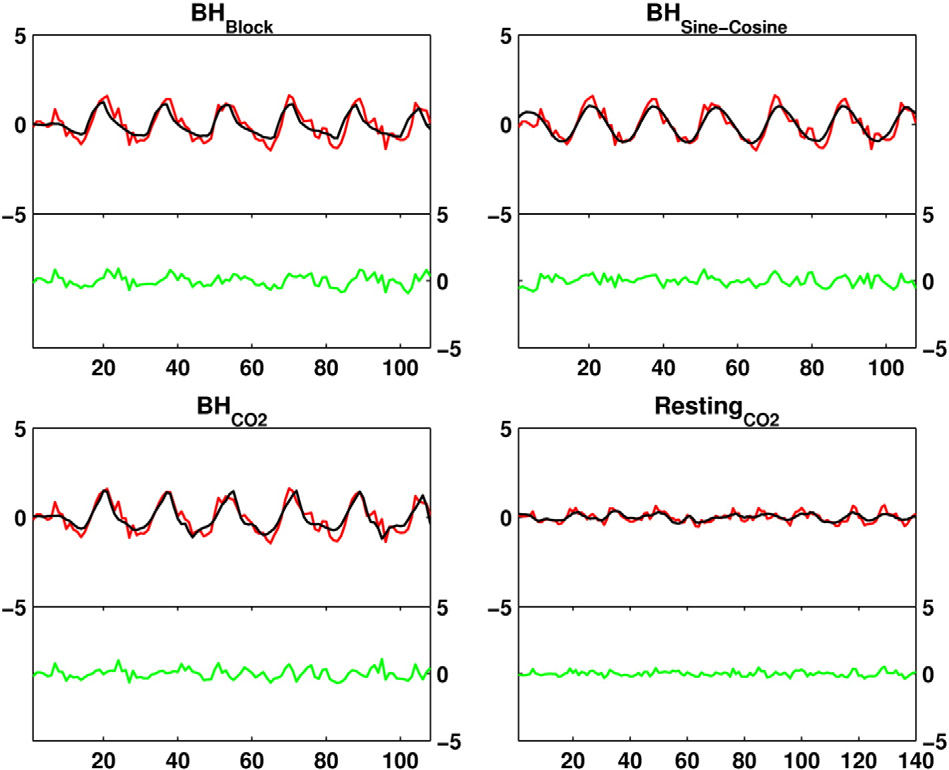
For each method an example of a model fit of an average participant is provided. The BOLD time series (averaged over the grey matter) is plotted in red, the model in black. Residuals are plotted in green.

An example for a model fit for each of the analysis methods is given in Fig. 3. Since the variance explained by each of the models is expected to differ between regions, the median model fit was extracted on a regional basis as well. In all of the regions, either the sine–cosine or the CO_2_ trace model explained the most variance (see Supplement Fig. 1).

### Estimations of vascular reactivity

Estimates of the median %BOLD/mm Hg CO_2_ throughout the grey matter differed between the three breath-hold based analysis methods (*χ* [2] = 19, *p* < .0001). Pairwise comparison revealed lower estimates for the *BH*_Block_ approach compared to the *BH*_Sine−cosine_ (*p* = .0006) and the *BH*_CO2_ (*p* = .004) approach. *BH*_Sine−cosine_ and *BH*_CO2_ regressors did not result in different vascular reactivity estimates (*p* > .30; all *p* values corrected for number of pairwise comparisons (3)). The vascular reactivity estimates in the *Resting*_CO2_ did not significantly differ from the *BH*_CO2_ approach (*χ* [1] = 1.14, *p* = .29). However, the *Resting*_CO2_ yielded significantly more variable values than the *BH*_CO2_ approach (F-test: *χ* [13] = 7.6, *p* < .001; also see Fig. 2b). This pattern holds true in most of the considered individual regions, and in a number of regions the *Resting*_CO2_ also resulted in significantly higher vascular reactivity estimates (see Supplement Fig. 2).

Estimations of the median extracted percent signal change/mm Hg CO_2_ in the grey matter showed high between-participant correlations with each other when the three breath-hold analysis approaches were used. The values obtained with the *Resting*_CO2_ were significantly correlated with the *BH*_CO2_, and with the *Resting*_RSFA_ method. Vascular reactivity estimates from the two temporal standard deviation approaches (*Resting*_RSFA_ and *Breath*_Tsd_) were also significantly correlated (Table 1). Scatter plots for all correlations are shown in Supplement Figs. 4 and 5. Correlations between *BH*_CO2_ and *Resting*_CO2_ for all investigated regions can be found in Supplement Table 1.

### Comparison of vascular reactivity maps obtained during breath-hold vs. resting state scans

**Fig. 4:**
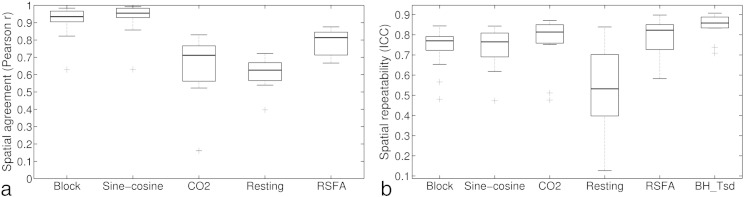
a) Spatial agreement between the vascular reactivity map using *BH*_CO2_ method and *Resting*_CO2_ method. As a comparison, agreement is also plotted between the maps using *BH*_CO2_ and the maps obtained with the *BH*_Block_ design and *BH*_Sine−cosine_ regressor. b) Comparison of spatial ICCs of vascular reactivity maps obtained with the six methods. ICCs were calculated over voxels in grey matter only.

In order to quantify agreement between the vascular reactivity maps produced using the breath-hold data vs. the resting state data, the *r* spatial values between the maps were obtained for session 1 for each person. Vascular reactivity maps created with the *BH*_Block_ and *BH*_Sine−cosine_ regressor showed good agreement with the maps created with the *BH*_CO2_ regressor in all participants (median *r* spatial > .92), whereas the maps created using the *Resting*_CO2_ showed more variable but on average high correspondence to the breath-hold (*BH*_CO2_) created map (median *r* spatial = .64, min. = .10, max. = .76). The *Resting*_RSFA_ data set showed similar agreement with the *BH*_CO2_ maps with a median *r* of .63 (min. = .40, max. = .72; Fig. 4a). For the resting state data, the *Resting*_CO2_ method and *Resting*_RSFA_ maps had good agreement (median *r* = .69, min. = .44, max. = .88). Maps created with the two standard deviation based measures (*Resting*_RSFA_ and *BH*_Tsd_) agreed very highly (median *r* = .85, min. = .70, max. = .89), as well as maps from the *BH*_CO2_ and *BH*_Tsd_ approaches (median *r* = .81, min. = .67, max. = .88).

If vascular reactivity can be measured with a resting state scan, day-to-day differences in vascular reactivity as measured by breath-holding should correspond to day-to-day differences in vascular reactivity as measured by the resting data. An *r* spatial was calculated for each person using a day-to-day difference map from *BH*_CO2_ and *Resting*_CO2_ data. The maps did not appear to be related, with a median *r* spatial of .14 (min. = .01, max = .37).

### Repeatability of vascular reactivity estimates

Repeatability for the median percent signal change extracted from grey matter was good using the *BH*_Block_ design (ICC = .71, *p* = .002), *BH*_Sine−cosine_ (ICC = .74, *p* < .001) and using the *BH*_CO2_ (ICC = .62, *p* = .007), and fair using the *Resting*_CO2_ data (ICC = .42, *p* = .06) but excellent for the *Resting*_RSFA_ data (ICC = .76, *p* < .001). Scatter plots are shown in Supplement Fig. 5. ICCs were also calculated on a regional level (see Fig. 6). In most of the regions, either the *BH*_Block_ or the *BH*_Sine−cosine_ and in some cases the *Resting*_RSFA_ data show the highest repeatability.

CVs_between_ for the median percent signal change extracted from grey matter were 31% for the *BH*_Block_ method, 26% for the *BH*_Sine−cosine_ method, 25% for the *BH*_CO2_ method and 60% for the *Resting*_CO2_, respectively. Median (iqr.) CVs_within_ for the four methods were: 11(23)% (*BH*_Block_), 12(12)% (*BH*_Sine−cosine_), 8(12)% (*BH*_CO2_) and 42(42)% (*Resting*_CO2_). Friedman tests revealed no significant differences in CVs_within_ between the three breath-hold based approaches (*χ* [2] = 1.7, *p*= .42), but a significant difference between the *BH*_CO2_ method and the *Resting*_CO2_ (*χ* [1] = 7.1, *p*= .008). CVs for all ROIs can be found in Supplement Tables 2–3 and Supplement Fig. 3.

Spatial repeatability was calculated for each person and each analysis method (see Fig. 4b). Spatial repeatability depended on the analysis method for the breath-hold scan (*χ* [2] = 9, *p*= .01). Pairwise comparisons revealed that highest repeatability was found for the *BH*_CO2_ (*BH*_CO2_ vs. *BH*_Block_: *p* = .032 (uncorrected), *BH*_CO2_ vs. *BH*_Sine−cosine_: *p* = .0075 (uncorrected); only difference between *BH*_CO2_ vs. *BH*_Sine−cosine_ would survive correction for number of comparisons (3)), with no significant difference between *BH*_Sine−cosine_ and *BH*_Block_ models (*p*(uncorrected) = .60). Repeatability was lower for the resting state data set than for *BH*_CO2_ (*χ* [1] = 14, *p*= .0002).

Spatial repeatability was also calculated for the temporal standard deviation approaches. Both approaches showed good to excellent repeatability in all participants (see Fig. 4b).

### How many cycles of breath-hold are needed to get reproducible vascular reactivity maps?

**Fig. 5:**
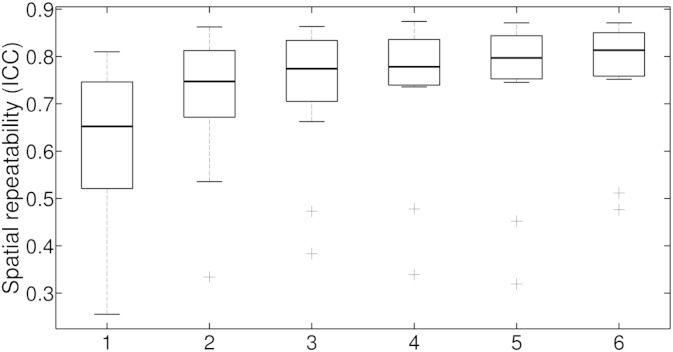
Spatial repeatability dependent on how many cycles of breath-hold are included in the analysis. Data for the CO_2_ trace regressor are presented.

**Fig. 6:**
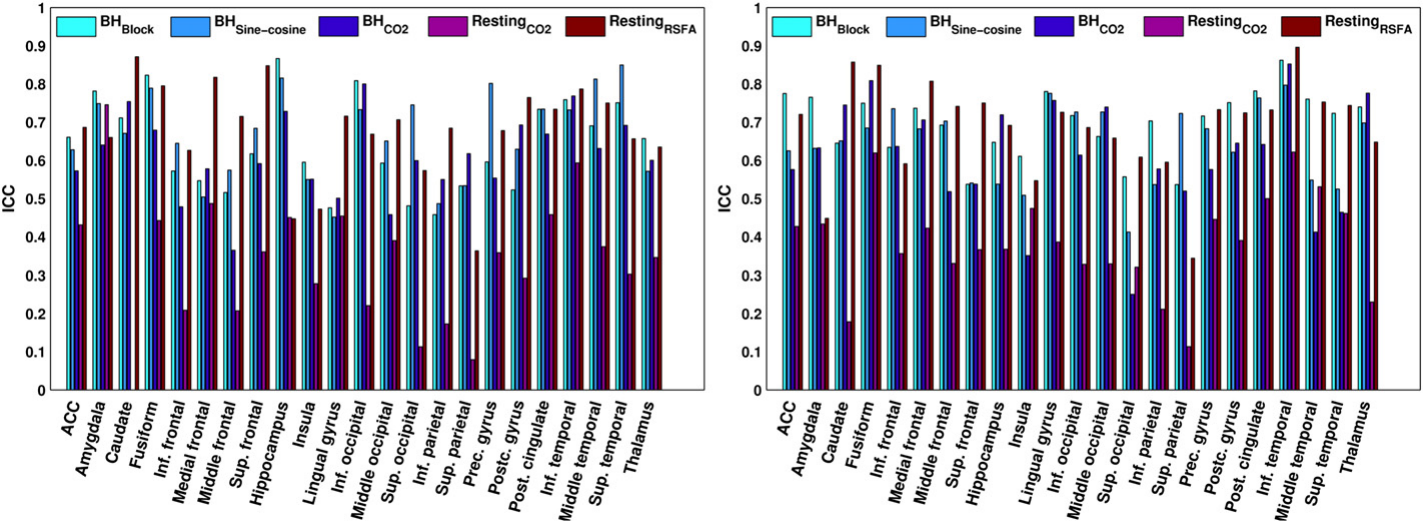
The repeatability (ICC) of the estimated vascular reactivity for each of the methods in each region of interest in the left hemisphere (left) and right hemisphere (right). An ICC of < .40 indicates “poor” repeatability, .40–.59 “fair” repeatability, .60–.74 “good” and > .74 “excellent” repeatability.

Repeatability was calculated for the maps obtained using different numbers of breath-hold cycles included in analysis. The number of cycles significantly influences the ICC_spatial_ values obtained (*χ* [5] = 39, *p* < .0001; see Fig. 5). Comparing subsequent numbers of cycles shows that implementing 2 cycles yields higher repeatability than 1 cycle (*p* = .0013), 3 higher repeatability than 2 (*p* = .0325), 4 higher repeatability than 3 (*p* = .0325), but there is no difference between 4 and 5 (*p* = .11), or five or six (*p* = .11), however, between four and six (*p* = .0075). Only the difference between 1 and 2, as well as 4 and 6 cycles survive correction for the number of comparisons (6).

### Repeatability of breath-hold performance

In order to examine performance differences between the two sessions, an ICC was calculated for each person over the time course of the CO_2_ regressor. A median of ICC = .81 (min. = 26, max. = .93) suggests that performance is highly repeatable in most participants (see Supplement Fig. 6a). The range of the physiological trace used to scale the BOLD response showed fair repeatability for the breath-hold based approach (ICC = .41, *p* = .06; see Supplement Fig. 6b) as well as for the resting based approach (ICC = .54, *p* = .02; see Supplement Fig. 6c), indicating that the extent of CO_2_ increase in session 1 only shows a fair relationship to the CO_2_ increase in session 2.

## Discussion

In this study, we compared three different ways of analysing a breath-hold scan (*BH*_Block_, *BH*_Sine−cosine_, and *BH*_CO2_) and an approach using resting state data set (*Resting*_CO2_) with regard to different aspects of measuring vascular reactivity: model fit of the regressors, repeatability of obtained vascular reactivity maps, and repeatability of regionally extracted vascular reactivity estimates. We compared vascular reactivity maps obtained during the breath-hold scan to the maps obtained during the resting scan to see whether resting state scans could be potential replacements for breath-holds when it comes to estimating vascular reactivity. Additionally, we extracted RSFA (*Resting*_*RSFA*_) and the temporal standard deviation of the breath-hold data (*BH*_Tsd_) set to make results directly comparable to previous studies, and to investigate the effects of modelling the data by using recorded CO_2_ traces.

### Model fit and estimations of vascular reactivity

The model fit of each regressor was assessed by calculating the amount of variance in the BOLD time series explained by the regressor. In grey matter, the *BH*_Block_ design accounted for significantly less variance than the *BH*_Sine−cosine_ or *BH*_CO2_ trace regressors, which on average explained about 40% of the BOLD time series (min. = 19%). These values are comparable with those previously reported by Murphy et al. (2011). In the resting state data, the CO_2_ regressor could on average only explain less than 20% of the variance (min. = 6%) which is also comparable to previous studies (Chang et al., 2009; Wise et al., 2004). Within the grey matter, the amount of variance explained by the models varies substantially between regions, with particularly good model fits in the frontal cortex, and poorer model fits in other regions such as the hippocampus and inferior temporal cortex. Lower model fits for the resting state data than for the breath-hold data were found in most of the considered regions. In other words, the BOLD time course during rest is not as strongly influenced by CO_2_-related physiological changes as it is during the breath-hold scan. This was expected since the change in CO_2_ during the breath-hold scan is stronger (median range in CO_2_: 8 mm Hg) than during a resting scan with no other task-instruction than to relax (median range in CO_2_: 2 mm Hg). The difference in model fit between breath-hold data and resting data suggests that the proportion of neuronally-driven fluctuations is higher in the resting state data, which makes it more challenging to measure vascular reactivity.

However, also for the breath-hold task the regressors are not sufficient to explain all variance in the BOLD time series. For this reason, in order to obtain a measure of vascular reactivity it is important to identify the CO_2_ related signals in the BOLD time course. Estimating vascular reactivity by simply using the temporal fluctuation of the BOLD time series, as suggested by Kannurpatti and Biswal (2008), gives a good indication of how much the BOLD signal fluctuates but disregards the influence of the source of fluctuation. In this study we used a different approach, by defining vascular reactivity as the amplitude of the model fitted data set divided by the CO_2_ change. This allowed one to specifically look at CO_2_-related BOLD fluctuations. In regions with high vascular reactivity, a good model fit was obtained. Using a *BH*_Block_, fit estimations of vascular reactivity were lower than for the *BH*_Sine−cosine_ and *BH*_CO2_ trace model. The vascular reactivity estimates in BOLD signal change/mm Hg CO_2_ values are comparable to previous studies (Kastrup et al., 2001; Liu et al., 2013). In fact, comparing vascular reactivity measures obtained this way to the measure of temporal standard deviation (*BH*_Tsd_) only yielded in medium sized correlations, even though both values were derived from the exact same scan.

The resting state analysis (*Resting*_CO2_) – for which model fit was lower than in any of the breath-hold based methods – provided the highest vascular reactivity estimates in all regions (however, the difference was only significant in a number of them). Vascular reactivity measures were highly variable between participants. In participants with lower model fit, lower vascular reactivity is estimated; but in participants for which the CO_2_ regressor explains a considerable amount of the BOLD time course, a small change in CO_2_ seems to result in a big change in BOLD, resulting in estimates of vascular reactivity about twice as high as the estimates obtained through breath-holding and higher than previously reported (Wise et al., 2004). Since the same participants were used in both scans, there must be another reason for this difference in estimation than actual differences in vascular reactivity. One explanation for that finding is that in the resting state scan CO_2_ fluctuations might be correlated with neuronal fluctuations in participants with these extremely high vascular reactivity estimates. This would result in a higher BOLD response than caused by the CO_2_ increase only. Confounded neural fluctuations might be related to arousal, which affects the breathing pattern and as a consequence the BOLD response. The over-estimation of vascular reactivity in some participants makes it difficult to interpret the findings for the resting state scan and suggests that breath-hold tasks cannot simply be replaced by resting state scans if adequate measures of vascular reactivity are the aim.

### Agreement of vascular reactivity measures and maps obtained during breath-hold vs. resting state scans

Kannurpatti and Biswal (2008) found that vascular reactivity maps obtained during a resting scan are very similar to the maps obtained during breath-hold scans, using the temporal standard deviation approach. We replicated this finding using the temporal standard deviations (*Resting*_*RSFA*_ and *BH*_Tsd_), however, using the CO_2_ modelling approach the agreement between breath-hold and resting state derived maps was considerably lower with a median correlation of .64, as was the correlation between the conventional breath-hold analysis *BH*_CO2_ and the *Resting*_*RSFA*_ (median of .63). This means that temporal standard deviations show some agreement with the CO_2_ modelled data, but are probably driven by more than changes in CO_2_.

Even if vascular reactivity maps derived with the suggested methods are comparable, in order to be able to replace breath-hold scans with resting state scans when measuring vascular reactivity, not only the spatial agreement of the resulting maps is required, but also between-participant correlations. This is because vascular reactivity measures are used to decrease between-participant variance. At the participant-level, we found a relationship between the extracted vascular reactivity in grey matter obtained through *BH*_CO2_ and the *Resting*_CO2_ values of *r* = .63. This is similar to what has been reported by Kannurpatti et al. (2012). In comparison, we found correlations of around > .80 between the three breath-hold based approaches (however, it has to be noted that these measures were all derived from the same scan). Vascular reactivity measured with the *Resting*_*RSFA*_ method did not correlate significantly with any of the breath-hold based approaches. Another study that looked at the relationship between RSFA and vascular reactivity (as measured by hypercapnia) in the whole-brain found a correlation of .36 (Liu et al., 2013). This correlation coefficient is similar to ours (*r* = .41), which did not reach significance with our sample size. However, the small effect size does indicate vascular reactivity assessed through breath-holding and RSFA are not interchangeable.

### Repeatability of vascular reactivity estimates and maps

Repeatability was estimated in order to obtain an indication for how reliable and stable the vascular reactivity measures are. Repeatability was calculated for the extracted vascular reactivity (%BOLD/mm Hg CO_2_) in all considered regions. In most regions, repeatability of breath-hold based vascular reactivity can be classified as good (.60–.75), while most of the ICCs obtained during the resting state data would be classified as poor or fair (< .40/< .60). Which breath-hold resulted in the highest repeatability was region-dependent, but in most regions the *BH*_CO2_ regressor resulted in lower repeatability than the *BH*_Block_ or *BH*_Sine−cosine_ regressors. This was the case even though the model fit for the *BH*_CO2_ trace regressor was found to be higher than for the *BH*_Block_ regressor and not different from the *BH*_Sine−cosine_ regressor. It is possible that by recording the CO_2_ trace during both sessions, additional measurement error was induced that resulted in slightly lower repeatability. These findings are in contrast with what Bright and Murphy (2013) reported, comparing a CO_2_-based approach with a HRF convolved ramp regressor. However, their study was specifically designed to induce variability in task-performance between participants. In our study most participants managed to hold their breath for 15 s without problems, which might be the reason why the CO_2_ analysis did not lead to higher repeatability than the other regressors. The vascular reactivity measures obtained with the *BH*_Block_ or *BH*_Sine−cosine_ regressor are influenced by performance, and performance turned out to be highly repeatable in most participants. This might have led to a boost in repeatability when either of these two regressors is used.

When looking at CV_within_ as an additional indicator of repeatability, no significant differences between the three breath-hold based approaches could be found. However, the *Resting*_CO2_ method resulted in significantly higher CV_within_ values than the *BH*_CO2_ method, again suggesting lower reproducibility of the vascular reactivity estimates using the resting state approach. While the ICC measure is not only reflective of the variation of estimates within participants over time, it also takes into account the variability between participants in the sample used for repeatability estimation. On the other hand, the CV_within_ only reflects the variability of the measurements within individuals, and is therefore a useful complementary measure. Our results suggest that the low ICC obtained for the *Resting*_CO2_ method is not due to reduced variability between participants, since the CV_between_ as well as the CV_within_ are in fact higher than for any of the other methods.

How repeatable the vascular reactivity measures were, varied from region to region. This reflects regional differences in the size and reliability of the vascular reactivity estimates (also reflected by regional differences in the model fit), as well as differences in the stability of vascular reactivity over time. Day-to-day differences in factors such as tiredness or caffeine intake might influence vascular reactivity more in some regions than in others. In some regions, such as the insular cortex (Banzett et al., 2000; Evans et al., 2002) the vascular reactivity measures are likely to be influenced by neural confounds, which might also lead to less reliable estimates of vascular reactivity. Indeed, the insular cortex was amongst the regions with the least repeatable estimates in this study.

*Resting*_*RSFA*_ appeared to be highly repeatable in most of the regarded regions. However, due to the low agreement with breath-hold based vascular reactivity measures, it is unclear what this method actually measures. During the breath-hold, the strong BOLD responses to the task can be assumed to be strongly driven by the arterial CO_2_ changes, even though neural aspects of breath-holding might still influence the BOLD signal change to a certain extent (e.g. Banzett et al., 2000, Evans et al., 2002). During the resting state scan, no such intense physiological changes are present. Resting state scans are widely used to assess cognitive networks, assuming the BOLD signal variations largely originate from fluctuations in neuronal activity rather than fluctuations in CBF associated with underlying non-metabolically demanding variations. Even after correcting for physiological changes (such as variations in breathing), neural networks can still be detected (for reviews see Birn, 2012; van den Heuvel and Hulshoff Pol, 2010). This means, that in the resting state data set neural aspects are likely to have a bigger influence on the BOLD signal than is the case during breath-hold scans. Using only the temporal standard deviation over the whole time series makes it impossible to study the source of this signal fluctuation, even though some of the variance can be explained by CO_2_ reactivity, as indicated by a medium between-participant correlation between *Resting*_*RSFA*_ and *Resting*_CO2_. Also, the amount of physiological BOLD fluctuations – irrespective of the underlying vascular reactivity – might vary between participants, reducing the interpretability of vascular reactivity in resting state data.

The change in CO_2_, which directly influences the vascular reactivity estimates, only shows fair repeatability, suggesting that there might be day-to-day differences in the CO_2_ increase during breath-holding. An additional analysis was performed to determine the repeatability if BOLD % signal changes are not divided by the change in CO_2_. ICCs increase for all analyses (*BH*_Block_: ICC = .90, *p* < .0001; *BH*_Sine−cosine_: ICC = .84, *p* < .0001; *BH*_CO2_: ICC = .77, *p* < .001; *Resting*_CO2_: ICC = .84, *p* < .0001) but most substantially for the *Resting*_CO2_ data set. This is another indicator for the possibility that the CO_2_ trace might be confounded with other factors leading to a boost in the BOLD response independent of the actual change in CO_2_. Interestingly, not dividing by the CO_2_ range also gave slightly better ICC values for the breath-hold related BOLD changes, which indicates that CO_2_ measurement error probably does play a role. In this particular sample breath-hold performance and CO_2_ changes might not have been variable enough to detect the benefit from recording and using the end-tidal CO_2_ trace as reported by Bright and Murphy (2013).

Since repeatability of vascular reactivity values (BOLD signal change/mm Hg CO_2_) is not only influenced by the quality of the method but also by possible day-to-day differences in vascular reactivity, spatial repeatability of the vascular reactivity maps might be a better indicator for reliability. Also, the performance of breath-hold will affect all voxels in the same way, and repeatability of the maps should be less dependent on day-to-day differences in performance. We found that the *BH*_Block_ design leads to lower mean spatial repeatability than the *BH*_Sine−cosine_ or *BH*_CO2_ trace regressors, but all median ICCs for the breath-hold data sets are above .75, which can be classified as excellent. Spatial repeatability of the vascular reactivity maps using resting state data was very variable between participants, with a median classified as good for the *Resting*_CO2_ and a median classified as excellent for the *Resting*_*RSFA*_ analysis. This means that vascular reactivity maps obtained with resting state data are repeatable in most but not all participants. The lower repeatability in some participants could result from session-to-session differences in what participants were doing during the resting state scans and from activation of different networks on each occasion. This again suggests that resting state data are harder to interpret when it comes to vascular reactivity. Looking at spatial repeatability of vascular reactivity maps of the breath-hold based approaches, two outliers had only fairly repeatable maps. Further exploration revealed that these were the two participants with the least variability in their vascular reactivity estimates over the brain.

#### Reliability versus validity

In this paper, we aimed to compare the repeatability of various measures of vascular reactivity. Repeatability consists of the two aspects reliability and temporal stability. A highly repeatable measure is likely to be a reliable measure. From all the methods we assessed, the *Resting*_*RSFA*_ gave the most repeatable results, suggesting it might be the most reliable method. However, it is not clear to what extent RSFA reflects fluctuation driven by vascular vs. neural factors. At the moment, the gold-standard of measuring vascular reactivity is introducing hypercapnia by CO_2_ challenges or breath-holding, even though these two methods also have neural confounds (Banzett et al., 2000; Evans et al., 2002) hard to control for. We showed that assessing vascular reactivity by modelling the breath-holding periods vs taking the temporal standard deviation in this data gives related but not highly correlated vascular reactivity measures (correlation between *BH*_Block/*Sine*−*cosine*/*CO*2_ and *BH*_Tsd_). Moreover, the highly repeatable *Resting*_*RSFA*_ scores did not significantly correlate with the modelled breath-hold data. If we assume that breath-holding is a valid measure of vascular reactivity, then *Resting*_*RSFA*_ is unlikely to be a good indicator for vascular reactivity.

#### How many cycles of breath-hold are needed to get reproducible vascular reactivity maps?

To answer the question how many cycles are necessary to obtain repeatable vascular reactivity, we analysed the BOLD time series several times, each time including a different number of breath-hold cycles. Only including 1 or 2 cycles of breath-holding does not result in repeatable maps in most participants. Based on our results, we recommend implementing at least 3 cycles to guarantee repeatable maps in all participants (but the two outliers), when a breath-hold duration of 15 s is used. It is possible that using longer duration less cycles are needed, while shorter duration more cycles might be necessary.

### Limitations and future directions

Even though we obtained good repeatability for regional vascular reactivity estimates, and high spatial repeatability of the maps, we need to be careful with the interpretation of the findings. Other factors than what we addressed as vascular reactivity – such as task performance – can play a role in the repeatability estimates. On the one hand, using the *BH*_Block_ design and *BH*_Sine−cosine_ regressors, task-performance plays a big role. If performance is bad, these regressors do not fit the data very well and lead to an unreliable vascular reactivity estimate. This problem is addressed by using the *BH*_CO2_ regressor, however, in our sample this regressor gave slightly lower repeatability values than the other two breath-hold regressors. In our sample, participants did not have problems performing the task, and the *BH*_CO2_ method is particularly useful for participants who have problems holding their breath (Bright and Murphy, 2013). In our study, overall good performance but slight variation between participants might have led to higher repeatability in the performance-dependent regressors.

Another problem that affects all three regressors is the fact that some neural confounds with breath-holding are likely to be present (Banzett et al., 2000; Evans et al., 2002). If this neural activity is repeatable, then vascular reactivity estimates will automatically show higher repeatability as well. Neural confounds are even more likely to affect the resting state scan, and we have shown over-estimation of vascular reactivity estimates that could be driven by neuronally driven fluctuations coinciding with CO_2_ fluctuations.

One difference in our estimates in resting state — data based vascular reactivity measures to previous attempts is that we used an eyes-open resting state scan rather than an eyes-closed scan. Eyes-open acquisition has been shown to yield more repeatable resting state networks but no difference in network strength (Patriat et al., 2013). McAvoy et al. (2008) reported higher frequency of CO_2_ fluctuations in the eyes-open vs. eyes-closed condition and Peng et al. (2013) found that the time lag between change in CO_2_ and BOLD response is shorter in the eyes closed condition than in the eyes open condition. However, they reported no significant difference in the model fit of the CO_2_ trace on the BOLD time series between the two conditions, indicating that both eyes open and eyes closed acquisition can be used to assess vascular reactivity. It is not clear whether the acquisition method (eyes open or closed) has an influence on the repeatability vascular reactivity estimates, and remains to be investigated.

## Conclusions

We found good repeatability of vascular reactivity estimates in most of the regions analysed and excellent repeatability of vascular reactivity maps, when the breath-hold scan is used for estimation. Also, at least three breath-hold cycles are necessary in order to obtain repeatable maps.

Analysing the resting state scan with a CO_2_ regressor in order to obtain vascular reactivity measures showed poorer model fit and repeatability than breath-holding. Also, vascular reactivity estimates in % change BOLD/mm Hg CO_2_ were considerably more variable with the resting state analysis, indicating the presence of some neural confounds in some participants. Vascular reactivity maps obtained with *Resting*_CO2_ and with *BH*_CO2_ revealed good spatial agreement but % change BOLD/mm Hg CO_2_ only showed moderate correlation, suggesting that it is not straight-forward to replace breath-holding with resting state scans in the assessment of vascular reactivity.

## Figures and Tables

**Table 1:** Between-participant Pearson correlation coefficients between the median vascular reactivity estimates (BOLD % signal change/mm Hg CO_2_) in grey matter obtained through the three breath-hold based approaches (*BH*_Block_, *BH*_Sine−cosine_ and *BH*_CO2_) and the resting state based approach (*Resting*_CO2_). Additionally, the two approaches based on the temporal standard deviation of the BOLD signal (*Resting*_RSFA_ and *BH*_Tsd_) were included to allow direct comparison to findings of previous studies.

	*BH*_Block_	*BH*_Sine−cosine_	*BH*_CO2_	*Resting*_CO2_	*Resting*_RSFA_	*BH*_Tsd_
*BH*_Block_	1	.93⁎⁎	.81⁎⁎	.47	.25	.44
*BH*_Sine−cosine_		1	.84⁎⁎	.39	.26	.54⁎
*BH*_CO2_			1	.63⁎	.41	.51
*Resting*_CO2_				1	.74⁎⁎	.52
*Resting*_RSFA_					1	.59⁎
*BH*_Tsd_						1

⁎ *p* < .05.

⁎⁎ *p* < .001.
