# Supplementary material for: Agreement and repeatability of vascular reactivity estimates based on a breath-hold task and a resting state scan
Source: Neuroimage. 2015 Jun;113:387–96. doi: 10.1016/j.neuroimage.2015.03.004 (PMC4441043; doi:10.1016/j.neuroimage.2015.03.004)
Supplement: Supplementary file 1 — Supplementary material. [file mmc1.pdf]

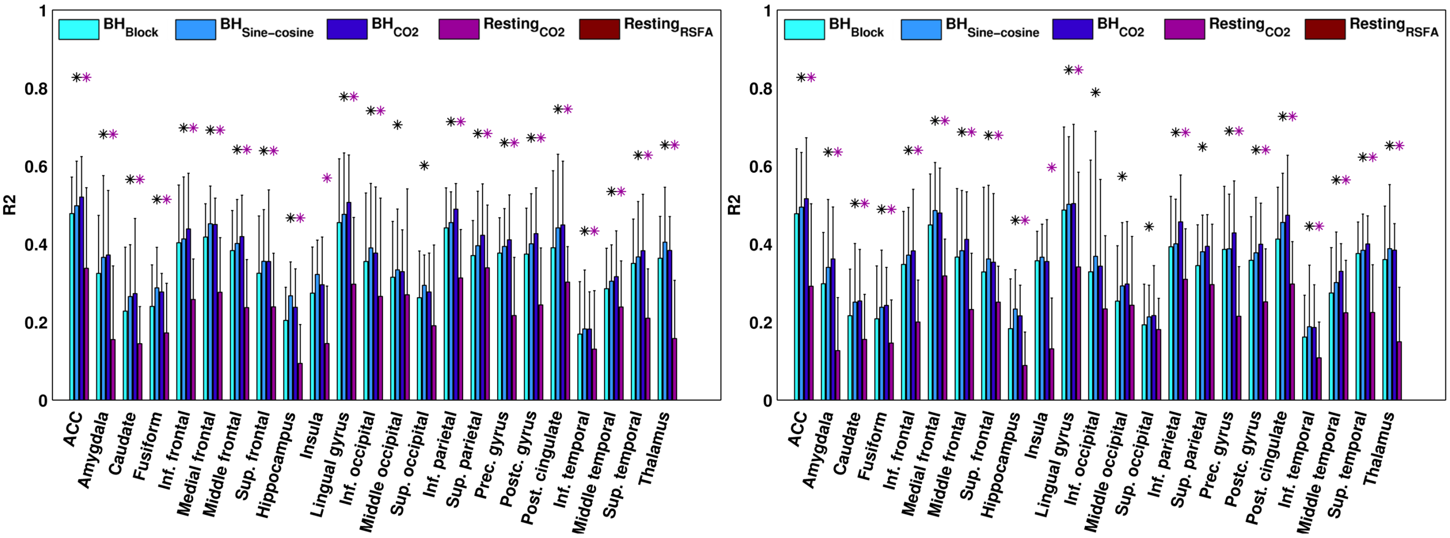

Figure S.1: The median amount of variance (error bars: interquartile range) explained by each of the methods in each region of interest in the left hemisphere (left) and right hemisphere (right). A black asterisk indicates a significant difference between the three breath-hold based analyses. A purple asterisk indicates a significant difference between the resting state based analysis and the CO_2 regressor based breath-hold based analysis.

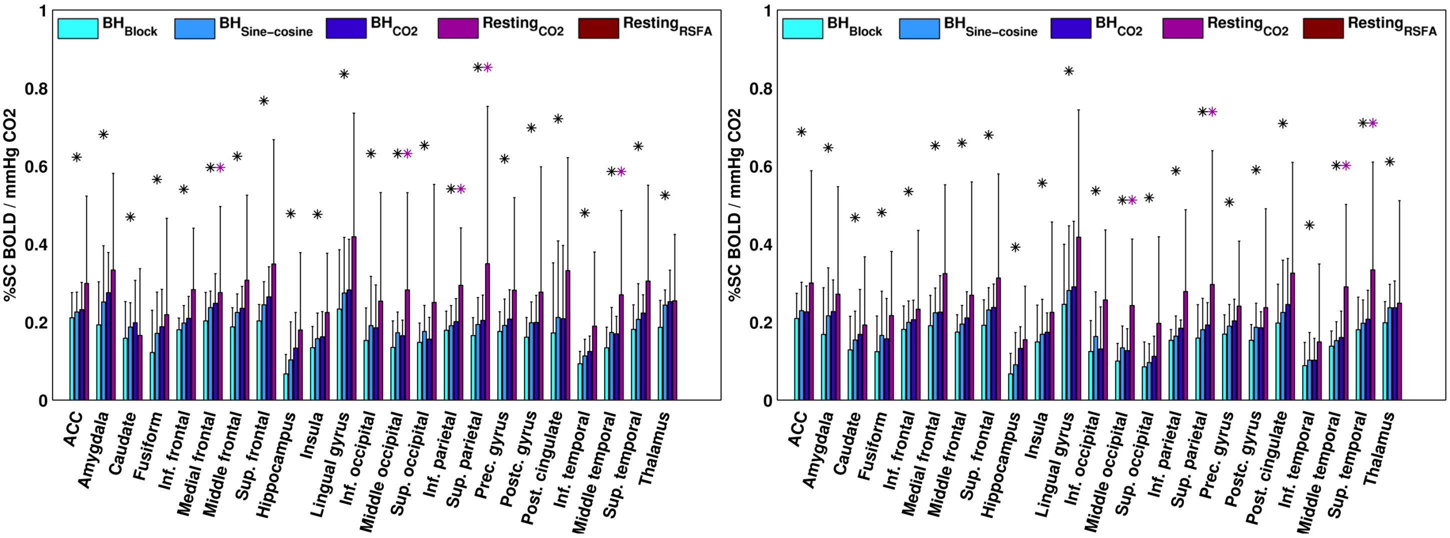

Figure S.2: The median estimated vascular reactivity (error bars: interquartile range) explained by each of the methods in each region of interest in the left hemisphere (left) and right hemisphere (right). A black asterisk indicates a significant difference between the three breath-hold based analyses. A purple asterisk indicates a significant difference between the resting state based analysis and the CO_2 regressor based breath-hold based analysis.

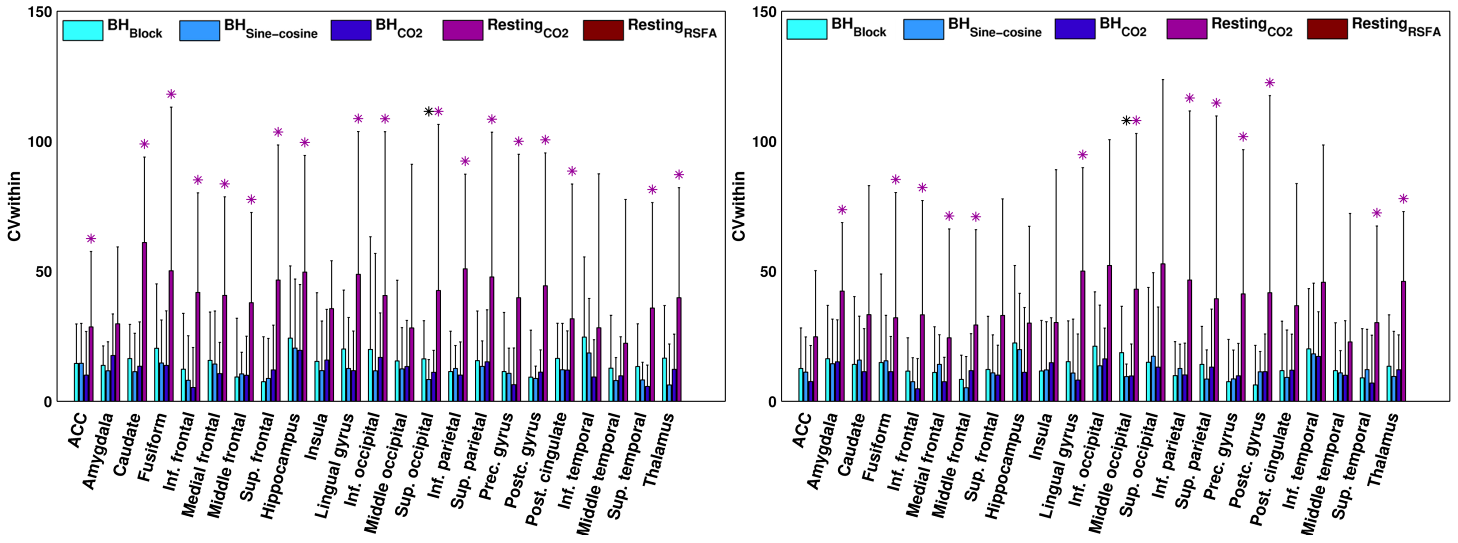

Figure S.3: The median estimated CV (error bars: iqr) by each of the methods in each region of interest in the left hemisphere (left) and right hemisphere (right). A black asterisk indicates a significant difference between the three breath-hold based analyses. A purple asterisk indicates a significant difference between the resting state based analysis and the CO_2 regressor based breath-hold based analysis.

Table S.1: Between-subject correlation of vascular reactivity values obtained through the BH_{CO2} and the $Resting_{\mathrm{CO2}}$ method, and as a comparison between-subject correlation of vascular reactivity values obtained through the $BH_{\text{sine-cosine}}$ and the BH_{CO2} method.

	BH_{CO2} and $Resting_{\mathrm{CO2}}$		$BH_{\text{Sine-cosine}}$ and BH_{CO2}	
Region	r	p	r	p
Left ACC	0.605	0.022	0.872	0.000
Left Amygdala	0.739	0.003	0.848	0.000
Left Caudate	0.280	0.333	0.866	0.000
Left Fusiform	0.655	0.011	0.826	0.000
Left inferior frontal	0.427	0.128	0.789	0.001
Left medial frontal	0.597	0.024	0.849	0.000
Left middle frontal	0.480	0.082	0.753	0.002
Left superior frontal	0.572	0.033	0.859	0.000
Left Hippocampus	0.632	0.015	0.808	0.000
Left Insula	0.347	0.225	0.826	0.000
Left lingual gyrus	0.468	0.092	0.887	0.000
Left inferior occipital	0.594	0.025	0.966	0.000
Left middle occipital	0.436	0.119	0.821	0.000
Left superior occipital	0.024	0.934	0.689	0.006
Left inferior parietal	0.262	0.365	0.797	0.001
Left superior parietal	0.469	0.090	0.894	0.000
Left precentral gyrus	0.361	0.205	0.827	0.000
Left postcentral gyrus	0.521	0.056	0.833	0.000
Left posterior cingulate	0.593	0.025	0.927	0.000
Left inferior temporal	0.565	0.035	0.917	0.000
Left middle temporal	0.540	0.046	0.795	0.001
Left superior temporal	0.572	0.033	0.803	0.001
Left Thalamus	0.539	0.047	0.917	0.000
Right ACC	0.379	0.181	0.910	0.000
Right Amygdala	0.401	0.156	0.888	0.000
Right Caudate	0.466	0.093	0.870	0.000
Right Fusiform	0.567	0.034	0.876	0.000
Right inferior frontal	0.521	0.056	0.778	0.001
Right medial frontal	0.519	0.057	0.833	0.000
Right middle frontal	0.411	0.144	0.744	0.002
Right superior frontal	0.711	0.004	0.812	0.000
Right Hippocampus	0.553	0.040	0.609	0.021
Right Insula	0.278	0.336	0.911	0.000
Right lingual gyrus	0.628	0.016	0.943	0.000
Right inferior occipital	0.374	0.187	0.946	0.000
Right middle occipital	0.270	0.351	0.811	0.000
Right superior occipital	0.320	0.265	0.943	0.000
Right inferior parietal	0.400	0.157	0.859	0.000
Right superior parietal	0.407	0.149	0.848	0.000
Right precentral gyrus	0.163	0.579	0.838	0.000
Right postcentral gyrus	0.276	0.340	0.899	0.000
Right posterior cingulate	0.426	0.128	0.891	0.000
Right inferior temporal	0.620	0.018	0.903	0.000
Right middle temporal	0.441	0.114	0.830	0.000
Right superior temporal	0.452	0.105	0.852	0.000
Right Thalamus	0.313	0.276	0.935	0.000

Table S.2: $CV_{between}$ for ROIs and the four analysis methods.

Region	BH_{Block}	$BH_{Sine\text{-}cosine}$	BH_{CO2}	$Resting_{CO2}$
Left ACC	31.97	27.13	25.62	59.61
Left Amygdala	44.05	35.33	30.58	71.24
Left Caudate	45.64	40.70	43.45	81.98
Left Fusiform	53.92	48.52	38.07	73.12
Left inferior frontal	30.30	24.14	23.59	60.73
Left medial frontal	33.63	27.39	25.00	61.43
Left middle frontal	28.90	24.33	23.85	55.32
Left superior frontal	31.66	26.82	27.23	60.64
Left Hippocampus	63.92	55.73	46.23	68.85
Left Insula	39.57	32.65	34.83	60.15
Left lingual gyrus	37.05	30.28	27.43	81.24
Left inferior occipital	65.21	58.45	53.86	84.28
Left middle occipital	41.07	33.31	26.36	75.37
Left superior occipital	27.85	28.06	23.51	76.11
Left inferior parietal	24.53	20.43	20.36	61.70
Left superior parietal	36.44	32.42	30.44	69.73
Left precentral gyrus	29.96	27.27	24.05	65.33
Left postcentral gyrus	26.65	23.69	22.07	66.50
Left posterior cingulate	48.32	40.88	41.45	69.82
Left inferior temporal	58.18	55.66	47.80	71.33
Left middle temporal	35.24	29.28	27.09	58.69
Left superior temporal	33.04	26.31	24.83	58.45
Left Thalamus	35.81	32.00	29.61	62.20
Right ACC	32.17	27.70	25.85	60.42
Right Amygdala	40.83	37.09	37.54	71.86
Right Caudate	42.10	36.76	37.21	67.16
Right Fusiform	53.65	49.87	38.46	81.51
Right inferior frontal	27.99	22.92	23.37	67.17
Right medial frontal	32.49	27.13	26.69	61.86
Right middle frontal	27.03	21.55	24.61	63.50
Right superior frontal	25.42	20.17	24.00	61.93
Right Hippocampus	53.99	47.94	40.58	65.13
Right Insula	27.64	26.33	28.66	65.93
Right lingual gyrus	44.72	37.43	36.32	82.30
Right inferior occipital	60.26	53.74	47.00	79.08
Right middle occipital	45.76	39.24	34.95	79.45
Right superior occipital	49.97	53.11	48.73	82.29
Right inferior parietal	26.11	21.17	23.41	67.16
Right superior parietal	29.49	24.59	29.90	75.32
Right precentral gyrus	26.14	24.03	24.44	73.93
Right postcentral gyrus	31.39	28.27	29.33	71.56
Right posterior cingulate	39.41	33.41	32.57	65.09
Right inferior temporal	64.01	55.65	54.61	86.57
Right middle temporal	30.42	25.44	26.79	60.46
Right superior temporal	29.67	24.28	24.50	60.11
Right Thalamus	38.67	33.54	35.00	60.41

Table S.3: CV_{within} for ROIs. p values refer to Friedman tests significance levels. BH comp.: comparison between BH based methods (BH_{Block}, $BH_{Sine-cosine}$, BH_{CO2}), CO_2 comp.: comparison between CO_2 based methods (BH_{CO2}, $Resting_{CO2}$).

Region	BH_{Block}	$BH_{\text{Sine-cosine}}$	BH_{CO2}	$Resting_{\text{CO2}}$	p (BH comp.)	p (CO2 comp.)
Left ACC	14.51(15.20)	14.58(15.37)	10.01(16.82)	28.56(28.98)	0.223	0.001
Left Amygdala	13.81(7.46)	11.68(11.18)	17.58(15.97)	29.76(29.56)	0.257	0.285
Left Caudate	16.40(13.10)	11.36(14.76)	13.47(17.04)	60.99(32.91)	0.607	0.008
Left Fusiform	20.39(24.73)	14.68(16.53)	13.76(21.01)	50.17(62.91)	0.751	0.033
Left inferior frontal	12.32(21.45)	8.04(17.10)	5.26(15.38)	41.83(38.29)	0.135	0.008
Left medial frontal	15.68(18.60)	14.28(20.37)	10.60(12.01)	40.69(37.89)	0.257	0.001
Left middle frontal	9.30(22.59)	10.51(8.32)	9.97(15.04)	37.86(34.69)	0.807	0.008
Left superior frontal	7.44(17.36)	8.76(15.37)	12.02(17.25)	46.58(51.98)	0.071	0.033
Left Hippocampus	24.27(27.77)	20.43(26.54)	19.56(25.35)	49.62(44.89)	0.931	0.008
Left Insula	15.34(26.37)	11.69(19.13)	15.81(19.50)	35.59(18.41)	0.257	0.285
Left lingual gyrus	20.07(22.66)	12.55(19.66)	11.79(15.16)	48.77(54.95)	0.168	0.001
Left inferior occipital	19.87(43.38)	11.68(45.20)	16.85(17.11)	40.66(62.99)	0.168	0.033
Left middle occipital	15.50(31.03)	12.43(15.84)	13.33(17.74)	28.19(62.92)	0.395	0.109
Left superior occipital	16.29(14.67)	8.37(7.58)	11.12(8.45)	42.56(63.93)	0.024	0.008
Left inferior parietal	11.40(15.48)	12.64(8.79)	10.09(12.65)	50.91(36.41)	0.751	0.008
Left superior parietal	15.60(19.06)	13.36(9.81)	15.19(19.97)	47.79(55.67)	0.257	0.008
Left precentral gyrus	11.41(22.72)	10.70(9.74)	6.35(14.12)	39.83(55.09)	0.931	0.001
Left postcentral gyrus	9.26(18.04)	8.75(4.76)	11.20(8.56)	44.40(51.10)	0.807	0.033
Left posterior cingulate	16.46(13.52)	12.07(17.88)	11.99(15.06)	31.66(51.84)	0.807	0.033
Left inferior temporal	24.65(30.83)	18.56(20.95)	9.34(14.26)	28.26(59.19)	0.424	0.285
Left middle temporal	12.72(20.33)	7.93(8.80)	9.77(15.01)	22.29(55.27)	0.395	0.109
Left superior temporal	13.32(16.44)	8.16(6.79)	5.65(8.22)	35.83(40.56)	0.607	0.008
Left Thalamus	16.56(20.24)	6.25(15.76)	12.24(13.54)	39.82(42.29)	0.135	0.033
Right ACC	12.58(15.61)	11.19(13.56)	7.55(13.88)	24.78(25.44)	1.000	0.109
Right Amygdala	16.35(20.49)	14.40(17.16)	15.17(16.07)	42.39(26.24)	0.931	0.008
Right Caudate	14.15(26.11)	15.79(16.88)	11.35(16.58)	33.28(49.59)	0.807	0.285
Right Fusiform	14.86(34.02)	15.51(17.53)	11.35(13.51)	32.04(48.22)	0.395	0.033
Right inferior frontal	11.57(12.80)	7.51(9.20)	4.76(11.63)	33.21(43.92)	0.257	0.033
Right medial frontal	11.06(17.54)	14.13(11.46)	7.47(9.43)	24.36(41.88)	0.607	0.033
Right middle frontal	8.43(9.26)	5.20(12.01)	11.71(14.23)	29.32(36.61)	0.395	0.008
Right superior frontal	12.19(20.44)	10.85(14.66)	10.06(11.49)	32.97(44.80)	0.395	0.109
Right Hippocampus	22.37(29.83)	19.85(21.68)	11.15(24.91)	30.04(37.20)	0.807	0.109
Right Insula	11.63(19.41)	12.04(18.53)	14.81(17.27)	30.24(58.73)	0.257	0.109
Right lingual gyrus	15.24(15.64)	10.84(20.76)	8.12(17.73)	50.05(39.73)	0.145	0.000
Right inferior occipital	21.15(20.91)	13.63(23.31)	16.28(11.83)	52.21(48.37)	0.089	0.109
Right middle occipital	18.66(17.84)	9.48(4.81)	9.68(12.35)	43.06(59.89)	0.010	0.033
Right superior occipital	14.95(28.77)	17.29(32.11)	13.12(23.10)	52.81(70.85)	0.931	0.109
Right inferior parietal	9.81(13.13)	12.62(9.35)	10.13(12.19)	46.60(65.02)	0.807	0.008
Right superior parietal	14.14(14.66)	8.51(11.21)	13.07(22.32)	39.39(70.31)	0.109	0.008
Right precentral gyrus	7.53(16.23)	8.59(11.10)	9.73(12.52)	41.26(55.51)	0.395	0.033
Right postcentral gyrus	6.27(15.20)	11.30(7.82)	11.36(14.47)	41.72(75.80)	0.424	0.033
Right posterior cingulate	11.72(19.08)	9.13(18.24)	11.94(13.86)	36.75(46.94)	0.607	0.285
Right inferior temporal	20.11(23.22)	18.20(27.19)	17.26(17.16)	45.71(52.84)	0.931	0.285
Right middle temporal	11.69(18.46)	10.86(8.58)	9.98(20.96)	22.79(49.45)	0.257	0.109
Right superior temporal	8.94(18.97)	12.12(15.51)	6.97(18.42)	30.22(37.19)	0.395	0.001
Right Thalamus	13.42(19.79)	9.54(17.29)	12.08(13.46)	46.10(26.81)	0.135	0.008

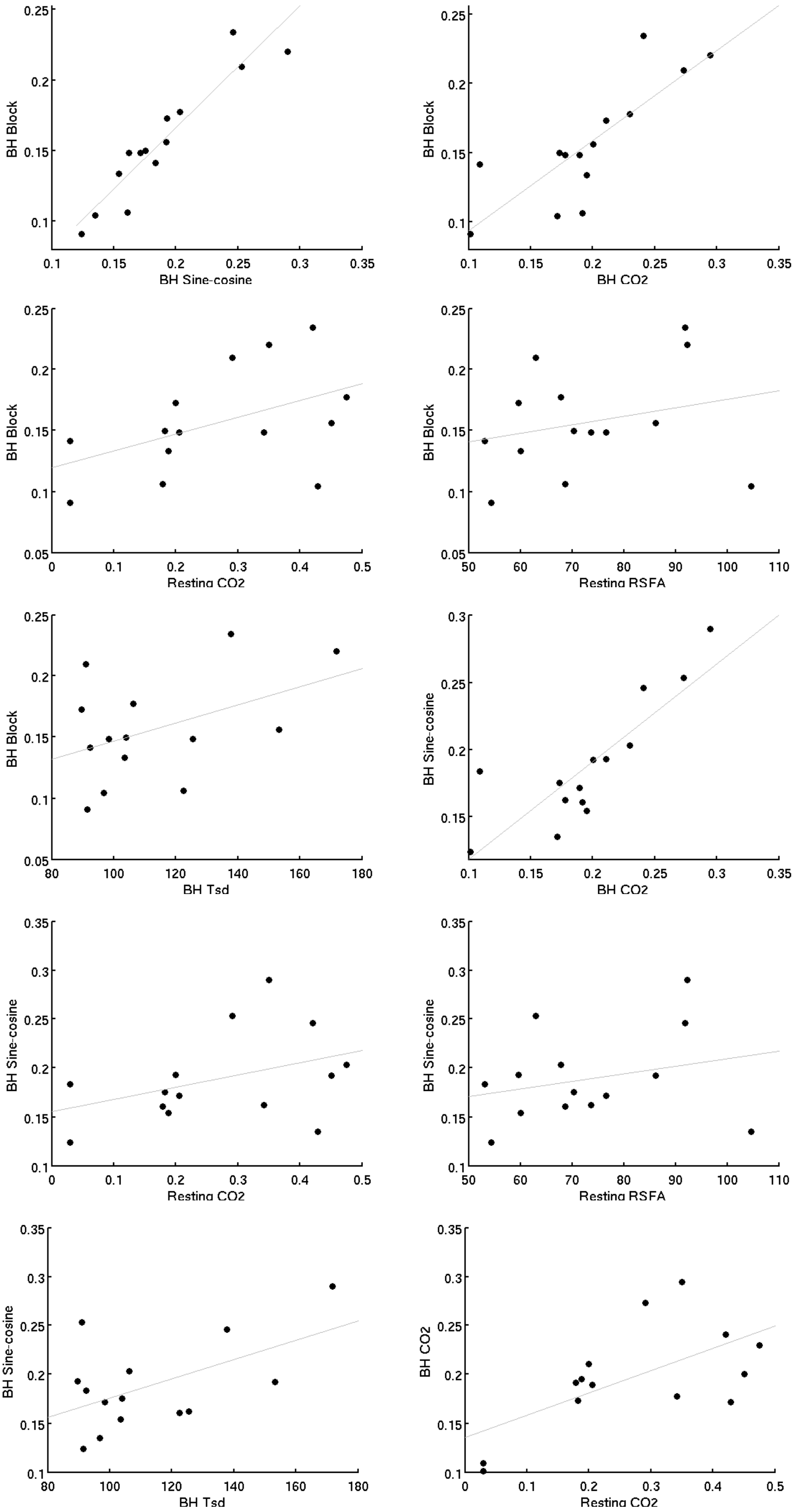

Figure S.4: Scatter plots for between-subject correlations of vascular reactivity estimates (averaged over gray matter) obtained by the six different methods BH_{Block},$\text{BH}_{\text{Sine-Cosine}}$, BH_{CO2}, $Resting_{\text{CO2}}$, $Resting_{\text{RSFA}}$ and BH_{Tsd}.

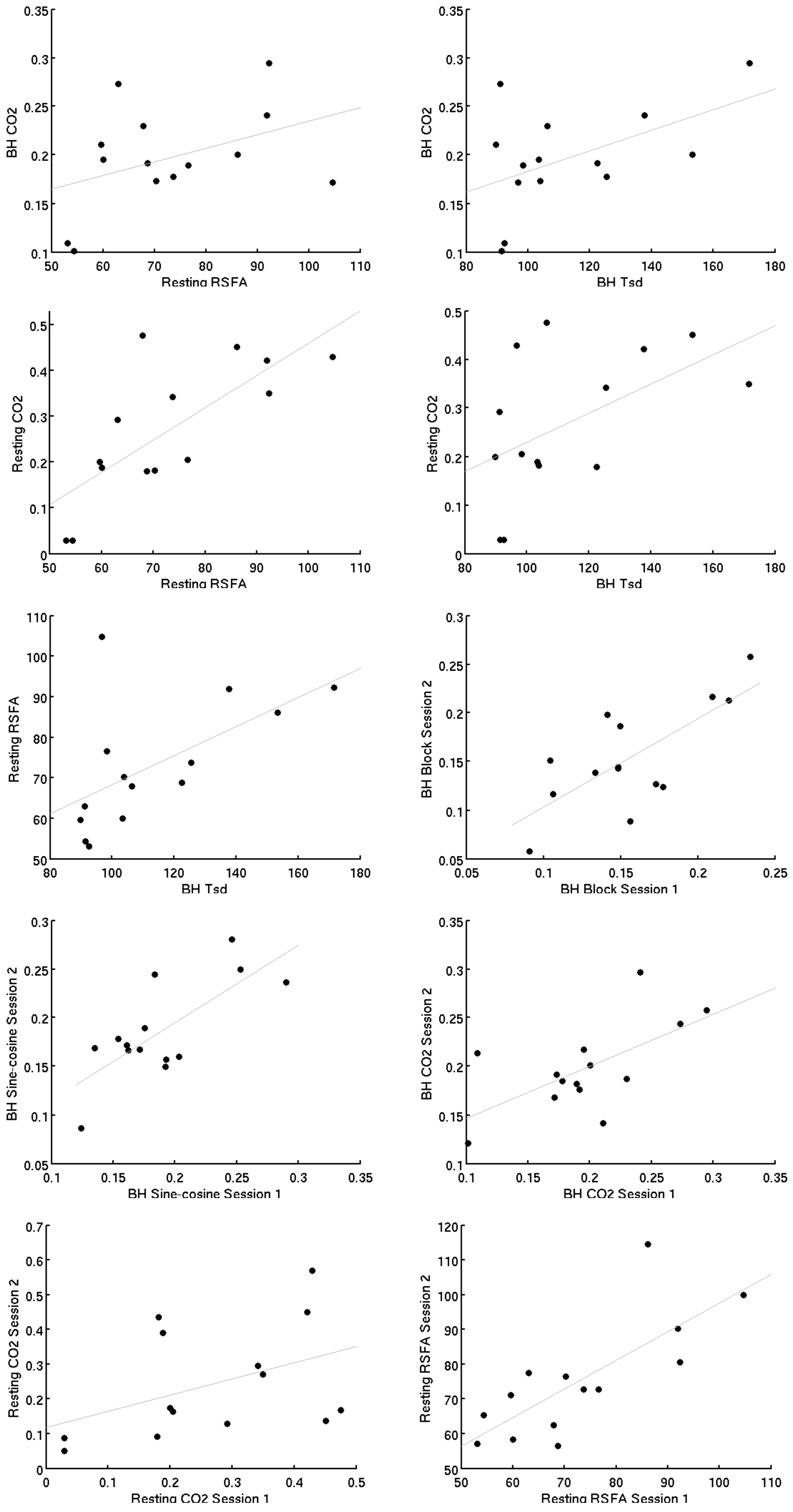

Figure S.5: Scatter plots for retest correlations of vascular reactivity estimates (averaged over gray matter) obtained by the six different methods BH_{Block},$\text{BH}_{\text{Sine-Cosine}}$, BH_{CO2}, $Resting_{\text{CO2}}$, $Resting_{\text{RSFA}}$ and BH_{Tsd}.

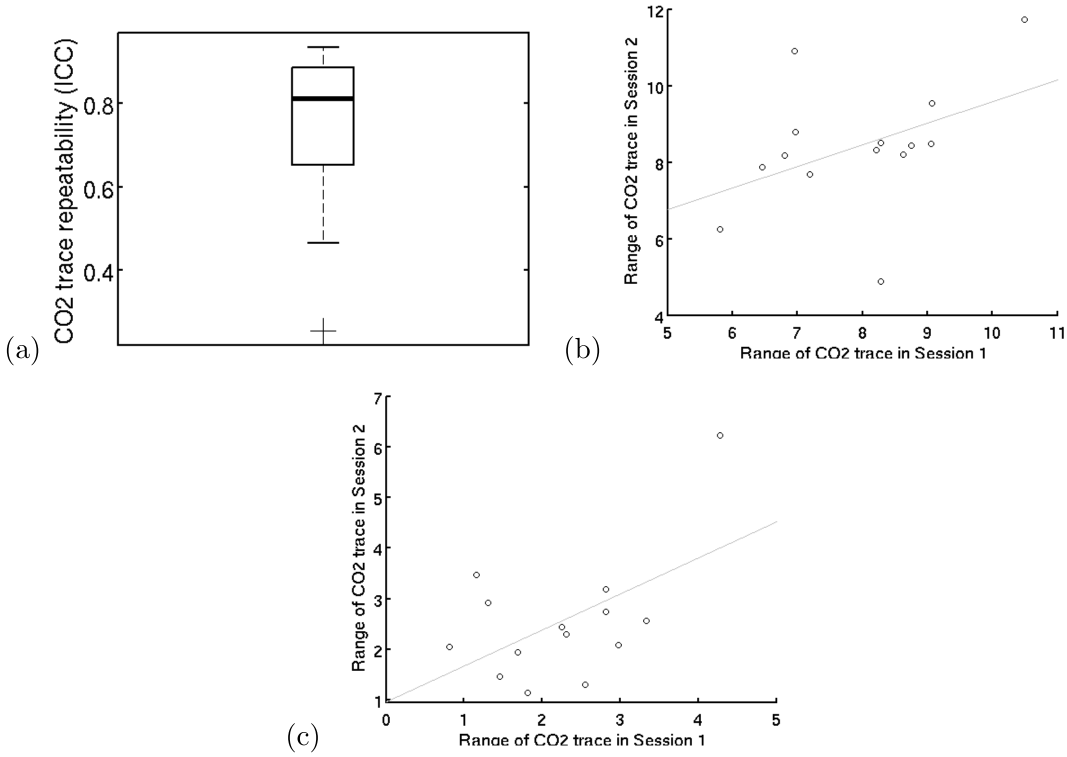

Figure S.6: a) Repeatability of the CO_2 trace during the breath-hold task. b) Scatterplot of the CO_2 range obtained during the breath-hold task in session 1 vs. in session 2. 3) Scatterplot of the CO_2 range obtained during the resting scan in session 1 vs. in session 2.
